# Extrafollicular CD19^low^CXCR5^−^CD11c^−^ double negative 3 (DN3) B cells are significantly associated with disease activity in females with systemic lupus erythematosus

**DOI:** 10.1016/j.jtauto.2024.100252

**Published:** 2024-10-09

**Authors:** Carlo Chizzolini, Jean-Charles Guery, Fanny Noulet, Lyssia Gruaz, Claire Cenac, Loredana Frasca, David Spoerl, Lionel Arlettaz, Alice Horisberger, Camillo Ribi, Stéphanie Hugues

**Affiliations:** a Department of Pathology and Immunology, Centre Médical Universitaire, School of Medicine, University of Geneva, Geneva, Switzerland; b Toulouse Institute for Infectious and Inflammatory Diseases (Infinity) INSERM UMR1291, CNRS UMR5051, University Paul Sabatier Toulouse, F-31024, Toulouse, France; c National Center for Global Health, Istituto Superiore di Sanità, Rome, Italy; d Clinical Immunology and Allergy, Department of Medicine, University Hospital and Faculty of Medicine, Geneva, Switzerland; e Department of Biology, ICH, Valais Hospital, Sion, Switzerland; f Service of Immunology and Allergy, Centre Hospitalier Universitaire Vaudois, Lausanne, Switzerland

**Keywords:** Systemic lupus erythematosus, Disease activity, B cells, Double negative B cells, Atypical memory, CXCR5, CD11c, TLR7, Extrafollicular response

## Abstract

**Objective:**

B cells play a major role in the development and maintenance of systemic lupus erythematosus (SLE). Double negative (DN) B cells defined by the lack of surface expression of IgD and CD27 have attracted recent interest for their sensitivity to Toll-like receptor 7 (TLR7) ligands and their potential role in the production of autoantibodies. Here we aimed at investigating the possible association of DN B cells and their subsets with SLE disease activity specifically in female patients, in which TLR7 gene has been reported to escape X chromosome inactivation.

**Methods:**

Peripheral blood mononuclear cells were purified from woman participating to the clinically well-characterized Swiss SLE Cohort Study (SSCS). PBMC from age-matched healthy females were used as controls. PBMC were stained for cell surface markers, intracellular Tbet and analyzed by multicolor cytofluorimetry. Single nucleotide TLR7 polymorphisms were assessed by polymerase chain reaction.

**Results:**

The median SLE disease activity index of the 86 females was 2, IQR [0–6], all but 8 were under chronic SLE treatment. B cells co-expressing CD11c and Tbet were increased, the mean fluorescence intensity (MFI) of CD19 was considerably reduced and we observed a large increase in CD11c + CXCR5-and CD11c-CXCR5-concomitantly with a reduction of CD11c-CXCR5+ B cells in SLE compared to 40 healthy donors (HD). When focusing on the DN B cell subset, we found a reduction of DN1 (CD11c-CXCR5+) and an increase of DN2 (CD11c + CXCR5-) and most impressively of DN3 (CD11c-CXCR5-) cells. The DN subset, particularly DN3, showed the lowest level of CD19 expression. Both DN1 and DN3 percentages as well as the CD19 MFI of DN cells were associated with SLE disease activity. The use of glucocorticoids, immunosuppressants, and antimalarials impacted differentially on the frequencies of DN B cell subsets. CD19 MFI in B cells and the percentage of DN3 were the strongest biomarkers of disease activity. The TLR7 snp3858384 G allele was associated with increased percentages of B cells and CD19^+^CD11c-CXCR5+ and decreased CD19^+^CD11c-CXCR5-.

**Conclusions:**

DN3 B cells are strongly associated with SLE clinical activity pointing to their potential involvement in disease pathogenesis, and CD19 expression level performs accurately as disease activity biomarker.

## Highlights

• Double negative (DN) B cells lacking the expression of CD11c and CXCR5 (DN3) are increased in SLE.
• B cells in general and DN3 in particular express lower levels of CD19 than controls.
• The frequency of DN3 B cells and CD19 expression level are in strong inverse correlation.
• The frequency of DN3 B cells has opposite association with the use of antimalarials and immunosuppressants.
• The frequency of DN3 B cell is strongly associated with SLE disease activity independently from treatment.

## Graphical abstract

**Figure:**
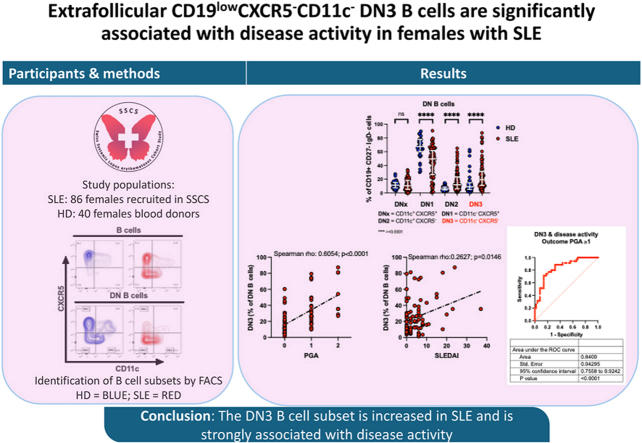

## 1 Introduction

Systemic lupus erythematosus (SLE) is characterized by profound B cell abnormalities, the production of autoantibodies (autoAb) and the generation of immune complexes contributing to immunopathogenesis. SLE preferentially affects females, which may be explained by the role of sexual hormones [1], the differential availability of transcription factors [2], as well as chromosomal disparities between females and males [3]. Notably, the escape from X-inactivation (XCI) in females may lead to increased copies of genes relevant to the immune response [4,5]. For instance, it has been shown that B cells from healthy woman with bi-allelic expression of toll-like receptor 7 (TLR7), carried by the X-chromosome, are more prone to TLR7-driven differentiation into plasmablasts or IgG class-switched B cells [6]. Interestingly, it was recently reported that dysregulated XCI in female mice led to reactivation of genes on the inactive X chromosome, including members of the TLR7-signaling pathway, resulting in B cell systemic autoimmunity [7]. Furthermore, X-inactive specific transcript (XIST) long noncoding RNA, specifically expressed in females to maintain X-chromosome balance, was recently shown to bind ribonucleoproteins which act as TLR7 ligands and promote autoimmune responses [8,9]. Interestingly, a particular B cell subset characterized by the absence of CD27 and IgD named double negative (DN) which frequency is increased in SLE [[10], [11], [12]] was shown to be particularly sensitive to TLR7 activation resulting in enhanced production of autoAb in SLE [13]. Additional characterization of DN B cells has led to the identification of DN B cells which may variably express CXCR5 and/or CD11c and categorized as DN1 (CD11c-CXCR5+), DN2 (CD11c + CXCR5-) or DN3 (CD11c-CXCR5-) [[13], [14], [15], [16], [17], [18]]. Parallel studies identified B cells co-expressing at their surface the myeloid marker CD11c and intracellularly the transcription factor Tbet which expression was driven by TLR7 [19,20]. These cells named ABC, also known as age-related B cells, autoimmune B cells, atypical memory B cells are expanded in murine models of lupus and SLE, in other systemic autoimmune diseases, as well as in chronic or acute infections [21,22]. The ABC subset exhibits tight similarities with the DN2 B cell subset [23].

Beside the function of TLR7 in the expansion of DN2/ABC B cell subsets enriched in SLE, evidence pointing to a relevant role of TLR7 in SLE pathogenesis stems from genetic data with polymorphisms associated with disease [24], and gain of function mutation identified in a female patient sufficient to induce lupus in mice [25]. In addition, the non-redundant role of TLR7 has been well established in lupus mouse models, and enhanced expression of TLR7 is sufficient to induce full blown autoimmunity and SLE-like disease in mice [26,27].

TLR-signals drive plasma cell differentiation and the development of pathogenic autoantibodies secreting cells through the extrafollicular pathway and the germinal center (GC) reactions during SLE [28]. We therefore assessed the frequencies of B cell subsets, including DN cells expressing or not CD11c or CXCR5 to discriminate between the extrafollicular and GC reaction in a well characterized cohort of females with SLE. Moreover, we examined correlations between the frequency of B cell subsets and clinical disease activity in the context of chronic treatment, and whether TLR7 polymorphisms were associated with these populations in our SLE female cohort. Confirming previous reports, we found that indeed ABC and DN B cells were increased in SLE, but in addition that the DN subset mostly associated with SLE disease activity was defined by the simultaneous absence of CD11c and CXCR5 (DN3). Further, we found that the reduced levels of CD19 expression was remarkably associated with disease activity and could act as disease biomarker in SLE females.

## 2 Methods

### 2.1 Cell donors

Our study population was composed of SLE individuals participating to the Swiss SLE Cohort Study (SSCS) [29] and satisfying the EULAR/ACR SLE classification criteria [30]. After informed, written consent, PBMC and clinical data were collected from all individuals participating to the annual SSCS visit. Male samples were then selected out, and the samples from 86 females, that were under standard of care were retained for further analysis. Their clinical characteristics are reported in Supplementary Table S1. Disease activity was evaluated according to physician global assessment on a 0 to 3 Likert scale and/or according to SELENA SLEDAI [31]. Age-matched, healthy females (HD) were platelet donors at the Blood transfusion center (Hôpitaux Universitaires de Genève, Switzerland). SSCS obtained ethic clearance in 2006 by « Commission Cantonale d'Ethique de la Recherche sur l'être humain (CCER 06–100), renewed in 2017 by Commission cantonale d'éthique de la recherche sur l'être humain (CER-VD 2017-01434). The ExplorX study obtained specific ethic clearance by « Commission Cantonale d'Ethique de la Recherche sur l'être humain (CCER 2021-01841).

### 2.2 Fluorescence flow cytometry analysis

PBMC from SLE and HD were collected and frozen in liquid nitrogen. Thawed PBMC were incubated with Viability Dye-eFluor 506 for 10 min at 4 °C, and after washing incubated with the Fc blocker (CD32; StemCell) for 15 min. After washing, CD19 - PE Vio615 (Miltenyi), CD14-PE (Immunotools), CD11c BV421 (BD), CXCR5-BB700 (BD), CD27 APC (BD), IgD FITC (BD) were added for 30 min at 2–8 °C. After fixation and permeabilization (Invitrogen), the cells were incubated with T-bet PECy7 (Ebiosciences) or isotype control, in permeabilization buffer for 30 min at RT. Events were acquired using LRS Fortessa (BD) and analyzed using Flow-Jo (10.10). To decrease the batch-to-batch variability in data acquisition, the FACS machine settings were adjusted each time using standardized calibration beads (Spherotech). Gating was performed upon selection of singlets and live cells (Fig. S1A).

### 2.3 Genomic DNA extraction and TLR7 SNP genotyping

Genomic DNA was extracted from PBMC using a NucleoSpin Tissue kit (Macherey-Nagel) or following overnight incubation of PBMC at 56 °C in lysis buffer (Tween 20, 0.05 %; NP 40, 0.05 % TrisHCl pH = 8, 10 mM; Proteinase K, 100 μg/ml). After 10 min incubation at 95 °C, samples were stored at −20 °C. Typing for the frequent di-allelic polymorphisms of *TLR7*, rs3853839 (NM_016562.3:c.∗881C > G) and rs179008 (NM_016562.3:c.32A > T), at the genomic DNA levels was performed using custom KASP competitive allele-specific PCR assays (LGC Genomics) run on a LightCycler 480 II instrument (Roche) as previously described [6].

### 2.4 Statistics

Continuous variables were summarized as median ± interquartile range and/or 95 % CI and analyzed using nonparametric tests including Mann-Whitney *U* test, Kruskal-Wallis test, or Spearman's rank order correlation (rho) where appropriate using Prism, 10.2 or SPSS version 27. Simple regression, multivariable linear and logistic regressions, receiving operator curves (ROC), were generated by using Prism 10.2. P values below 0.05 were considered significant.

## 3 Results

### 3.1 The frequencies of CD19, CD14 and nonCD19-non CD14 differ in SLE and HD PBMC

We assessed by flow cytofluorimetic analysis the proportions of CD19, CD14, and non-CD19-nonCD14 cell populations in 86 samples from SLE females and 40 HD. The gating strategy is shown in Supplementary Figs. S1A–B. Compared to HD, B cells percentages were lower in SLE (median 7.22 %, 95%CI [6.75–8.35] in SLE vs 9.91 %, 95%CI [8.54–10.75] in HD, p = 0.007). Of the 86 SLE, 13 were under B cell-depleting therapy (12 belimumab, 1 rituximab), which affected the total number and the percentage of B cells (Figs. S1B–D). When these 13 SLE were excluded, the percentage of B cells did not statistically differ from that of HD (SLE = 7.79, 95%CI [7.51–10.36], vs HD 9.91, 95%CI [8.54–10.75]; p = 0.091) (Fig S1 B, C). The largest numerical differences between SLE and HD were observed in CD14^+^ and nonCD19-nonCD14 cell populations with lower proportion of nonCD19-nonCD14 (64.1 %, 95%CI [60.3–67.1] in SLE vs 71.6 % 95%CI [68.7–73.8] in HD, p < 0.0001) and a higher proportion of CD14^+^ cells in SLE (28.55 % 95%CI [24.4–32.10] in SLE vs 17.95 % 95CI [16.6–20.6], in HD, p < 0.0001) (Fig. S1E). Thus, in this cohort of SLE females the proportions of major PBMC subsets greatly differed when compared to HD females.

### 3.2 Increased frequency of CD11c + T-bet + ABC in SLE patients

We first quantified the relative proportions of Naïve (IgD + CD27^−^), unswitched memory (USWM: IgD + CD27^+^), switched memory (SWM: IgD-CD27^+^), double negative (DN: IgD-CD27^−^) B cell subsets in SLE and HD (Fig. 1A). No statistical differences were observed in the proportions of naïve and SWM B cells subsets compared to HD. Consistently with previous reports [13], we found in SLE females an increased proportion of DN B cells (SLE 21.2 %, 95%CI [18.3–23.8]; vs HD 14.0, 95%CI [11.5-16-5]; p < 0.001) and a decreased proportion of USWM B cells (SLE 2.26 %, 95%CI [1.76–2.67]; vs HD 7 %, 95%CI [[6], [7], [8], [9]]; p < 0.001) (Fig. 1B).

**Fig. 1:**
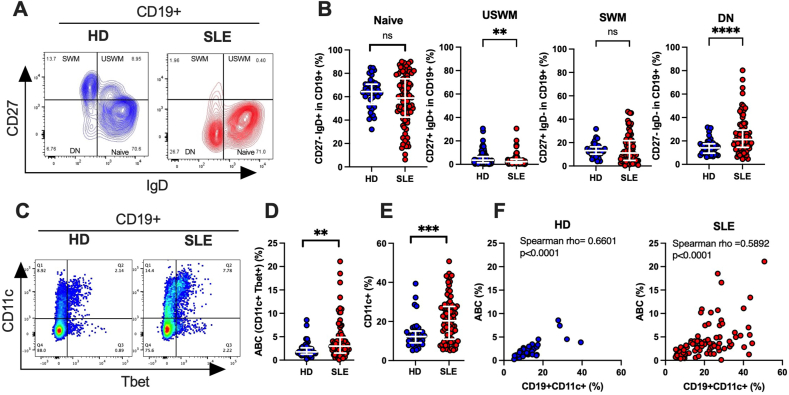
Double negative and ABC B-cell subsets are increased in SLE. CD19^+^ B cells were identified as naïve (CD27-IgD+), unswitched memory (USWM) (CD27+IgD+), switched memory (SWM) (CD27+IgD-), double negative (DN) (CD27-IgD-) and as ABC (CD11c + Tbet+) subsets by cytofluorimetry. **A**) Representative FACS contour plots used to identify the four subsets. Blue: HD; Red: SLE. **B**) Individual values, medians and interquartile range are reported for Naïve, USWM, SWM and DN subsets in CD19^+^ B cells. **C**) Representative FACS plots used to identify the ABC subset as double positive for CD11c and Tbet in CD19^+^ cells. **D**) Individual values, medians and interquartile range are reported for the ABC subset and **E**) total CD11c + cells of 100 % CD19^+^ B cells. **F**) Relationship between the percentages of ABC and CD11c + subsets in HD and SLE CD19^+^ B cells. In all panels: blue: HD; red: SLE. Comparisons between groups were made by Mann-Whitney *U* test. P values are ∗∗: <0.01, ∗∗∗: <0.001; ∗∗∗∗: <0.0001. Correlation assessed by Spearman rho. (For interpretation of the references to color in this figure legend, the reader is referred to the Web version of this article.)

The co-expression of CD11c and the transcription factor Tbet identifies a subset of B cells associated with autoimmunity named ABC [19,20]. Consistently with previous reports we found that in SLE ABC were significantly more frequent than in HD (Fig. 1C and D). This was also observed for the frequency of total CD11c + B cells (Fig. 1E). The frequency of CD11c+ in DN cells was much higher in SLE than in HD and to some extent in the naïve B cell subsets, much less or not at all in SWM and USWM (Fig. S2). Remarkably, despite substantial correlation (Fig. 1F) the frequency of CD11c + B cells largely exceeded the frequency of ABC cells more so in SLE than in HD. Indeed, the frequency of B cells expressing CD11c+ in the absence of Tbet (CD19^+^CD11c + Tbet-), was substantially more frequent in SLE (median 15.21 %, 95%CI [13.04–19.00]) than in HD (10.28 %, 95%CI [8.63–11.15], p = 0.002). All together and as expected from previous work [22,32], we observed an upregulated frequency of CD11c + T-bet + ABC in our female SLE cohort compared to HD. In addition, we showed that in SLE more than in HD, CD11c expression in B cells is largely independent form the expression of Tbet.

### 3.3 Expression of CXCR5 and CD11c on B cells define subsets with different proportions in SLE and HD

The expression of CXCR5 may participate to B-cell entry in B-cell follicles and germinal centers in secondary lymphoid tissues [28]. To categorize IgD-CD27^−^double negative (DN) subsets we used CXCR5 and CD11c as described [13]. While in lupus two DN populations were originally described DN1 CXCR5+CD11c- and DN2 CXCR5-CD11c+, additional DN3 CXCR5-CD11c-have also been described in COVID-19 [15], SLE [16] and autoimmune fibrosis [18]. When assessing B cells expressing or not CD11c and CXCR5 (Fig. 2A) we found that CD11c + CXCR5-and CD11c-CXCR5-were significantly more frequent in SLE than in HD, while CD11c-CXCR5+ cells were more frequent in HD than in SLE, with no significant differences in the CD11c + CXCR5+ subset (Fig. 2B). Similarly, when focusing on DN B cells (CD19^+^CD27-IgD-), SLE had significantly less DN1 (CD11c-CXCR5+) and significantly more DN2 (CD11c + CXCR5-) and DN3 (CD11c-CXCR5-) than HD (Fig. 2C). Not surprisingly, in both SLE and HD the frequency of DN2 correlated, but not perfectly, with the frequency of ABC (Fig. 2D and E). Overall, the variable expression of CD11c and CXCR5 by B cells highlight substantial differences when SLE are compared to HD and the larger frequency of B cells negative for CXCR5 may indicate that SLE B cells and in particular DN B cells are enriched in extrafollicular B cell subsets.

**Fig. 2:**
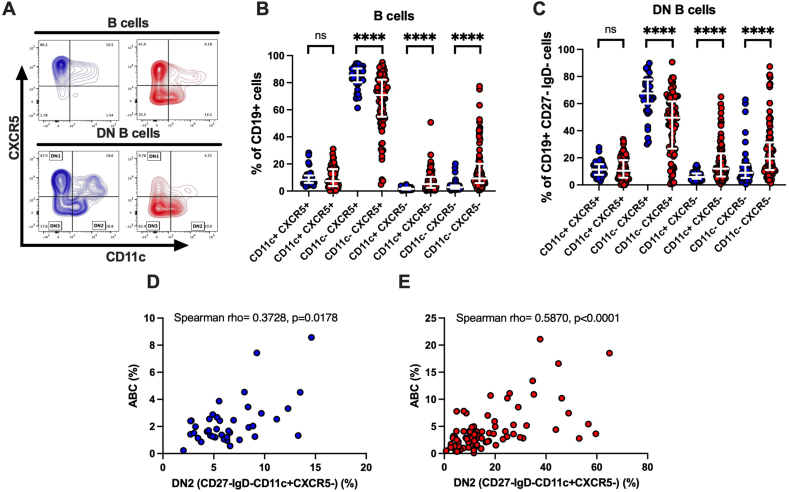
CD11c + CXCR5-, CD11c-CXCR5- DN2, DN3 B cells are increased in SLE. **A**) Representative FACS contour plots used to identify the expression of CXCR5 and CD11c on CD19 + B cells and on DN (CD27-IgD-) CD19^+^ B cells**. B, C**) Percentages of CD11c + CXCR5+, CD11c + CXCR5-, CD11c-CXCR5+, and CD11c-CXCR5-subsets identified by FACS analysis in B cells (**B**) and double negative (DN) B cells (**C**). In DN B cells the CD11c-CXCR5+ identify the DN1, the CD11c + CXCR5-the DN2, the CD11c-CXCR5-the DN3 subset. Individual values, medians and interquartile range are reported. CD11c-CXCR5+ are decreased and CD11c-CXCR5+, and CD11c-CXCR5-are increased in SLE compared to HD. **D)** HD and **E**) SLE correlation assessed by Spearman rho among the frequency of ABC and the DN2 subsets. Blue circles: HD. Red circles: SLE. Comparisons between groups were made by Mann-Whitney *U* test. ∗∗∗∗ = p < 0.0001. (For interpretation of the references to color in this figure legend, the reader is referred to the Web version of this article.)

### 3.4 The expression level of CD19 distinguishes SLE from HD and inversely correlates with DN3 CD11c- CXCR5- B cell frequency

CD19 participates in many ways to B cell development and responses to antigens and cytokines. In agreement with previous reports [32,33], we found that CD19 expression level was lower in SLE compared to HD (Fig. 3A). We assessed the expression level of CD19 in Naïve, USWM, SWM, and DN B cell subsets (Fig. S3), and found that compared to HD, CD19 had a significantly lower expression (lower MFI) in all B cell subsets (p < 0.001) (Fig. 3B). Although a moderate to high positive correlation of CD19 MFI was observed intra-individually among B cell subsets in both SLE and HD (not shown), CD19 MFI was significantly different across subsets (Kruskal-Wallis test p < 0.0001 in both SLE and HD), with the DN subset having the lowest and the USWM the highest expression level (Fig. 3B). These data confirm the lower *ex vivo* expression level of CD19 in SLE B cells, compared to HD, and indicate that lower CD19 expression is a robust marker of SLE B cells shared by all B cell subsets examined.

**Fig. 3:**
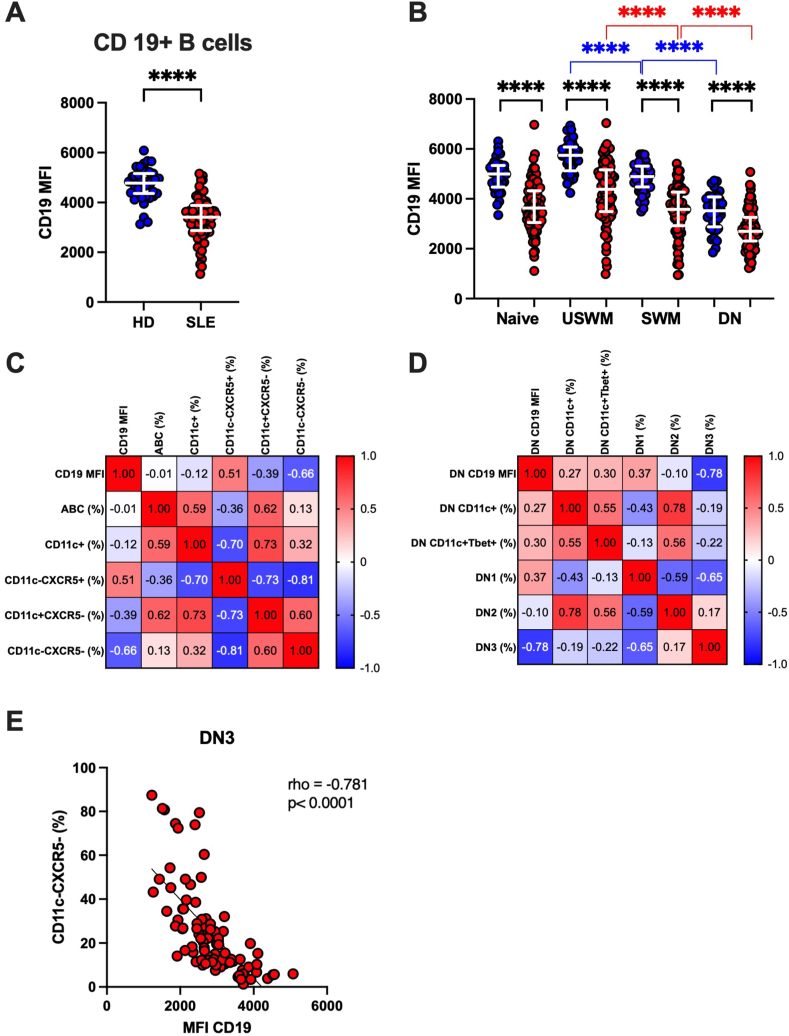
The mean fluorescence intensity (MFI) is decreased in B cell and B cell subsets and correlates strongly with CXCR5 expression in SLE. **A**) CD19MFI in B cells. **B**) CD19 MFI in naïve, USWM, SWM, and DN (Q4) B cells. CD19 MFI quantified as geometric mean. Individual values, medians and interquartile range are reported. In all subsets the CD19 MFI was lower in SLE than in HD. CD19 MFI was the lowest in DN B cells and the highest in USWM B cells. ∗∗∗∗ = p < 0.0001 for comparisons by Mann-Whitney *U* test. Differences amongst groups were highly significant within HD and within SLE by Kruskal-Wallis test (p < 0.0001). **C**) Correlation matrix of CD19 MFI within B cell subsets. **D**) correlation matrix of CD19 MFI within DN B cell subsets. Note that the strongest negative correlation was with the CD11c-CXCR5- B cells and the DN3 subset shown in **E**. The numbers in the squares are the respective Spearman's rho.

Given the observed distinct differences in CD19 MFI and in the proportions of CD11c + CXCR5-, CD11c-CXCR5+, and CD11c-CXR5- B cells particularly in the DN subset when SLE were compared with HD, we tested whether we could identify relations between these markers focusing on SLE samples. Strong negative correlations were observed between CD19 MFI and the frequencies of CD11c-CXCR5- B cells, while a positive correlation was observed with CD11c-CXCR5+ B cells (Fig. 3C). Interestingly, no clear correlation was found between CD19 MFI and the ABC and CD11c + CXCR5- B cell subsets (Fig. 3C). Similarly, when this analysis was conducted separately for the DN B cell population, the strongest association was the inverse correlation with DN3 subset (rho = −0.781, p < 0.0001, Fig. 3D, E and Fig. S3). Of interest, CD19MFI was in weak positive correlation with DN CD11c+, DN CD11c + Tbet+, and the DN1 subsets, and weak negative correlation with the DN2 subset. Overall, these data indicate that lower CD19 MFI was mostly associated with the expansion of extrafollicular CXCR5 negative B cells, including the DN3 subset.

Taking into consideration the effect of B-cell depleting therapies on the frequency and number of B cells (Fig. S1D), we tested whether there were statistically significant differences when B cell subsets from 12 SLE patients under chronic belimumab therapy were compared to the 73 SLE patients not under such treatment. Supplementary Table 2, shows that no differences in the frequency of DN cells and all the B cell subsets as defined by the presence or absence of CD11c, Tbet, CXCR5 as well as by CD19 MFI, were observed. These results justify the use of all samples in the analyses made to assess the relationship between B cell subset frequencies and disease activity and treatment.

### 3.5 B cell subsets and genetic polymorphisms

Although recently debated [34], genetic association studies have highlighted the contribution of TLR7 polymorphisms to SLE [24]. In particular, snp3853839 has been shown to enhance TLR7 expression and enhance B cell activation [35,36]. We assessed the allelic frequencies of snp rs3853839 G/C and snp rs179008 A/T in our study population and did not find any significant difference in their distribution between SLE and HD (not shown). In contrast, the G allele of snp rs3853839 was associated with significantly greater frequencies of B cells accompanied by increased CD11c-CXCR5+ and decreased frequencies of CD11c-CXCR5- B cell subsets in SLE. These associations were robustly present in the whole SLE population as well as when restricting the analysis to individuals not treated with B-cell depleting agents (Fig. 4A and B). Notably, we did not find significant differences in CD11c+ and CD11c + Tbet + subsets according to G/C polymorphism (not shown); nor robust associations were found for any of the B cell subsets here investigated with snp rs179008 A/T (Fig. 4A and B).

**Fig. 4:**
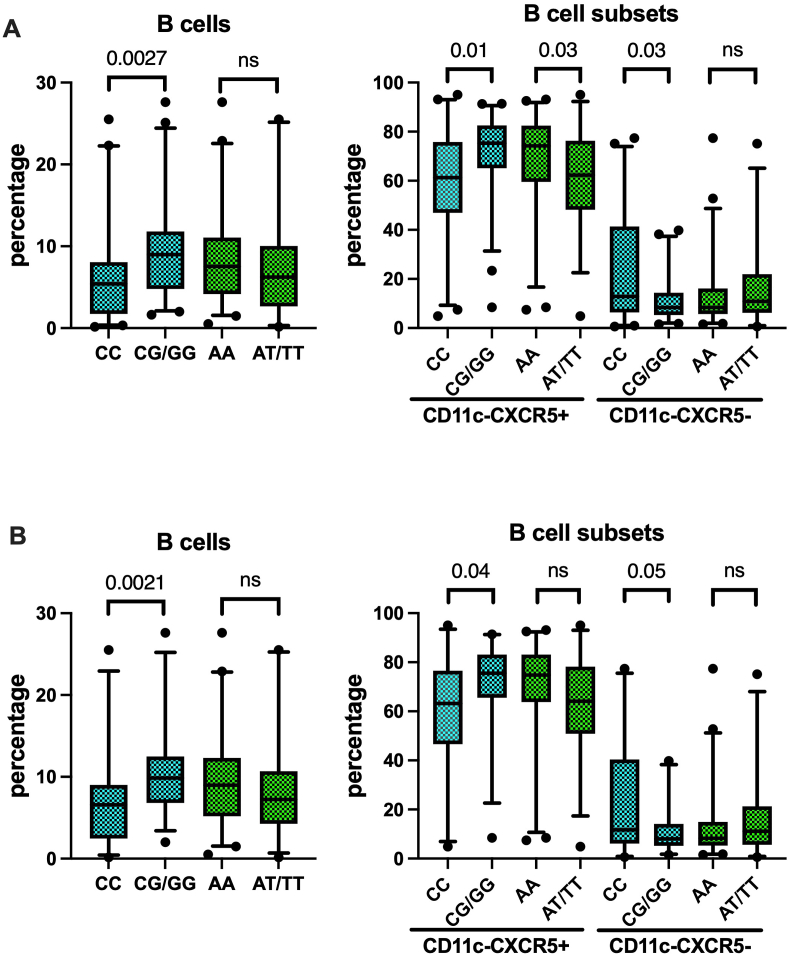
Single nucleotide polymorphism rs3853839 G/C but not rs179008 A/T is associated with increased frequencies of CD19^+^ B cells and CD11c-CXCR5+ B-cell subset. SNP identified by PCR as reported in material and methods. B cells and B cell subsets identified by FACS as described in the legend of Fig. 2. **A**) Data from all the included females (n = 86). **B**) Data from females under B cell therapy are excluded (n = 73). Box plots showing the median and the 25–75 percentiles. The whiskers identify the 5–95 percentiles. Black dots identify individual values. Comparisons between groups were made by Mann-Whitney *U* test. P values reported in the figure.

### 3.6 Increased frequency of circulating DN3 B cell subsets is associated with SLE disease activity

Focusing on the DN compartment, we then tested whether disease activity could impact on B cell subsets frequencies and CD19 MFI, thus potentially explaining the differences observed when SLE and HD were compared. Indeed, global SLE disease activity evaluated by PGA or SLEDAI was statistically associated with increased frequency of DN3, the reciprocal decreased frequency of DN1, and the associated decreased CD19 MFI in DN (Fig. 5A–D, Table 1A). No statistical association was observed with the frequency of DN2 B cells. Similar relationships with disease activity were observed for the ABC, CD11c-CXCR5+, CD11c-CXCR5-, and CD11c + CXCR5- B cell subsets as well as for CD19MFI in B cells (Fig. S4).

**Fig. 5:**
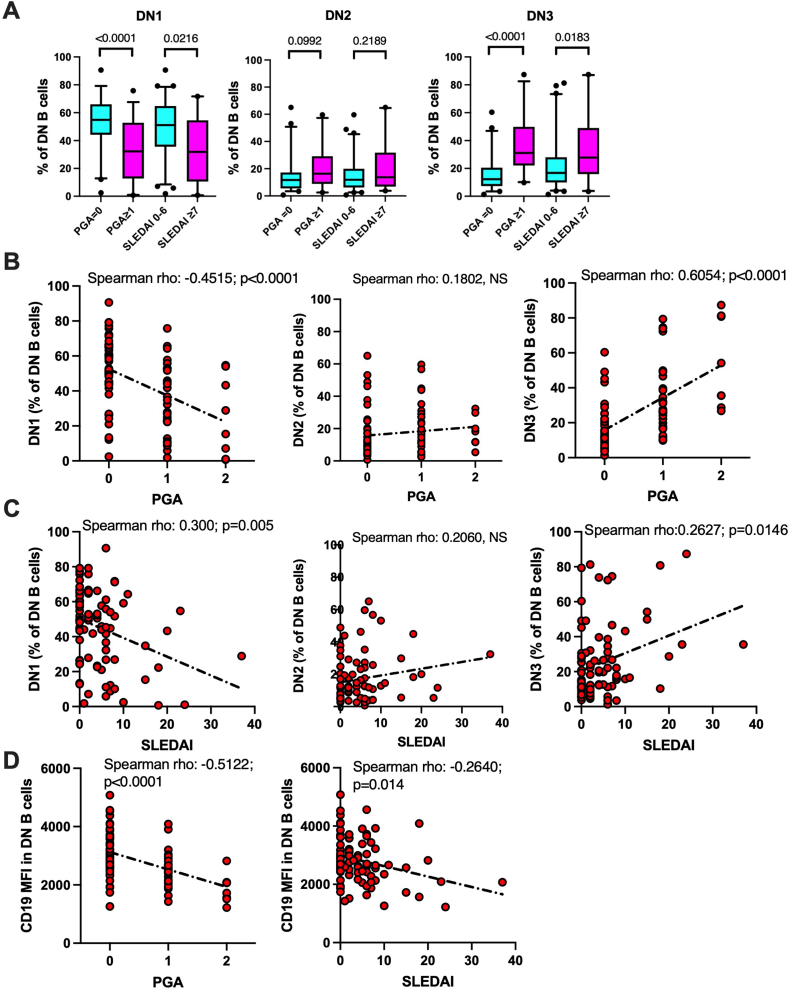
Double negative (DN) B cells subsets frequencies and CD19MFI are associated with disease activity. Subsets identified according to FACS as depicted in Fig. 2A. **A**) PGA: Physician global assessment; dichotomized: 0 = inactive; ≥1 = active; SLEDAI: SLE disease activity index dichotomized 0–6 inactive/low activity; ≥7 = active disease. Box plots showing the median and the 25–75 percentiles. The whiskers identify the 5–95 percentiles. Black dots identify individual values. Comparisons between groups were made by Mann-Whitney *U* test. **B, C, D**) PGA = 0: inactive; 1, moderately active, 2: active disease. SLEDAI: activity assessed according to SELENA. **D**) CD19 MFI quantified as geometric mean. Each circle represents individual values. Correlation computed according to Spearman rho. The dotted line represents simple linear regression.

**Table 1:** **Associations between double negative B cell subsets frequencies or CD19 MFI and disease activity and treatment. A**) Unadjusted, crude data. **B**) Data adjusted by multivariable analysis for age, ethnicity, and treatment**.** Reported are the P values computed by Mann-Whitney *U* test when groups were compared. PGA (dic) identify individuals with PGA = 0 inactive disease vs PGA = ≥1 active disease. SLEDAI (dic) identify individuals with SLEDAI 0–6 (inactive disease) vs SLEDAI ≥7 (active disease). In the treatment groups were compared individuals receiving vs those not receiving the treatment. GC: glucocorticoids, AM: antimalarial agents; IS: immunosuppressants. PGA: Physician global assessment; SLEDAI: SLE disease activity index assessed according to SELENA.

	A: Not corrected				B: Corrected for age, ethnicity, therapy			
	DN1	DN2	DN3	CD19MFI (DN)	DN1	DN2	DN3	CD19MFI (DN)
PGA (dic)	<0.0001	0.099	<0.0001	<0.0001	0.005	0.633	<0.0001	<0.0001
SLEDAI (dic)	0.022	0.216	0.019	0.011	0.066	0.713	0.088	0.027
Use of GC	0.051	0.199	0.112	0.237	0.724	0.59	0.422	0.784
Use of AM	0.002	0.015	0.049	0.124	0.695	0.783	0.919	0.807
Use of IS	0.007	0.255	0.029	0.201	0.919	0.298	0.183	0.778

We further tested whether treatment could impact on the frequencies of B cell and B cell subsets (Fig. S5). This was the case with immunosuppressants (IS) (which also included B-cell depleting agents) being associated with lower DN1 frequencies and increased frequencies of DN3, while antimalarials (AM) being associated with increased frequency of DN1 and decreased frequencies of DN2 and DN3, and glucocorticoids (GC) having no statistically significant effects on B cell subset composition. Thus, B cell subset frequencies appear to be affected by treatment with opposite relationships when IS and AM are compared. Interestingly, CD19 MFI in DN was not statistically affected by treatment (Table 1A; Fig. S5). In the attempt to distinguish between the overlapping effects of disease activity and treatments on the frequencies of DN B cells subsets we performed two distinct analyses. In the one hand, we tested DN subset frequencies in inactive versus active disease, with or without treatment (Fig. S6). In the other hand, we corrected for the impact of treatment – as well as for the effect of age and ethnicity - on B cell subsets frequencies by using multivariate analysis (Table 1B). Concordantly, these analyses indicated that higher frequencies of DN3 and the reciprocal decrease in DN1, as well as lower CD19 MFI were associated with disease activity assessed by PGA (and trendily by SLEDAI), independently of the effect of treatment. Notably, the data indicated that the DN2 subset was not robustly associated with disease activity also when correcting for the effect of treatment. To further dwell on the relationship between B cell subsets and disease activity, we calculated the area under the ROC curve (AUC) by logistic regression and found that the variables most highly and significantly associated with disease activity assessed as PGA or SLEDAI in the DN subset were the DN3 subset (Table 2 and Fig. S7) and CD19 MFI (Table 2). The joint use of both variables did not increase the AUC, while DN2 were not significantly associated. Similar results were obtained when the subsets in the whole B cells (not only in DN) were tested for prediction, the two variables best associated were the CD11c-CXCR5-subset and CD19 MFI (not shown).

**Table 2:** **Logistic regression model of SLE disease activity based on the frequency ofdouble negative (DN)B cell subsets and/or CD19MFI**. PGA: Physician global assessment; SLEDAI: SLE disease activity index assessed according to SELENA.

Outcome: PGA≥1	DN3	CD19MFI	DN3 and CD19MFI	DN1	DN2
ROC area	0.84	0.7791	0.8349	0.7591	0.6057
95 % CI	0.758 to 0.924	0.678 to 0.879	0.749 to 0.920	0.656 to 0.862	0.480 to 0.731
P value	<0.0001	<0.0001	<0.0001	<0.0001	0.0985
**Outcome: SLEDAI≥7**	**DN3**	**CD19MFI**	**DN3 and CD19MFI**	**DN1**	**DN2**
ROC area	0.6739	0.689	0.6864	0.6693	0.653
95 % CI	0.538 to 0.809	0.547 to 0.830	0.546 to 0.826	0.525 to 0.812	0.499 to 0.806
P value	0.019	0.0108	0.0119	0.0223	0.0389

Overall, the B cells subsets here studied were differentially associated with disease activity, with the DN3 and CD19MFI having the strongest associations and improving AUC the most.

## 4 Discussion

Profound alterations in the composition of B cell subsets characterize the peripheral blood compartment in SLE. In agreement with several previous reports [[10], [11], [12],^19^,^20^,^37^,^38^], we found that, compared to HD, DN B cells - considered antigen-experienced - were increased in our cohort of woman with SLE. Further, by exploring the expression of CD11c and CXCR5 on B cells and B cell subsets we found a substantial decrease in the frequency of cells positive for CXCR5, in particular but not only in the DN B cell subset in which most impressively the CD11c-CXCR5- (DN3) subtype was highly increased and associated with SLE disease activity. Finally, the CD19 MFI, which was in strong positive correlation with the frequency of cells positive for CXCR5, was substantially lower in SLE B cells and B cell subsets and was associated as well with SLE disease activity. The novelty here resides in highlighting the increased frequency of CD11c-CXCR5- B cells and DN3 B cells in SLE and the strong association of the percentage of DN3 B cells and of CD19 MFI with SLE disease activity.

The Dörner's group previously stressed heterogenous CD19 MFI in relationship with CXCR5 expression and subdivided B cell populations according to low, intermediate, and high CD19 expression describing the novel subset CXCR5-CD19^lo^ enriched in SLE in both SWM and DN B cells [16]. This adds to earlier descriptions of reduced expression levels of CD19 in SLE [32,33,39,40], and to the CXCR5-CD19^hi^ or CXCR5-CD19^int^ cells identified by the Santz group as increased in SLE [13]. Compared to CD19^hi^ and CD19^int^, the CD19^lo^ B cells were less responsive to activation by B cell receptor cross-linking as assessed by Syk phosphorylation [16], complementing previous findings showing that low CD19 expression (in association with low CD21) in SLE was associated with B-cell intrinsic impairment of TLR9 responses [40]. Our results on CD19 expression levels stress its reduction in SLE compared to HD in all major B cell subsets including, naïve, USWM, SWM, and particularly in DN and expand previous data. The question arises of the mechanisms resulting in lower CD19 expression in all SLE B cell subsets including naïve cells, which hints to SLE-specific B cell differentiation/maturation altered processes. The strong negative correlation of CD19 MFI with the percentages of CD11c-CXCR5- B cells and DN3 cells - suggesting shared mechanisms resulting in the loss (or absence) of CXCR5 and decreased CD19 expression - need to be explored.

The ABC cell subset has attracted large interest in recent years for its involvement in autoimmunity and infectious diseases [21]. However, inhomogeneity in the definition of the subsets as well as the clinical heterogeneity of SLE have contributed to discrepancies in the reported results. Several of our findings related to this B cell population need to be stressed. First, we found an increased frequency of CD11c + B cells irrespective of the positivity for Tbet when SLE were compared to HD, which suggests that other factors in addition to Th1-associated cytokines and signals mediated by TLR7 and TLR9 innate receptors thought to be involved in Tbet expression may be at play, particularly in SLE [14,41,42] and most importantly, suggest that the CD11c + B cell compartment can be heterogenous as shown in functional terms in murine SLE [43]. Second, at variance with previous reports [13,[44], [45], [46], [47]], the ABC as well as the related DN CD11c + Tbet+ and DN2 subsets, while increased when compared to HD, were not robustly associated with SLE disease activity as assessed by PGA and SLEDAI in our cohort. This may be related to the relatively low sensitivity of this B cell population confronted with the clinical characteristics of our SLE cohort and contrasts with the sensitivity to the clinics of the frequencies of DN1 and DN3, and CD19MFI. Indeed, the presence or absence of CXCR5 jointly with the absence of CD11c in total B cells and DN B cells was strongly associated with SLE clinical activity in our cohort. Compared to previous studies, we paid major attention to the impact of treatment on B cell subset distribution. We found that antimalarials and immunosuppressants had reciprocal differential associations with the frequencies of DN1 and DN3 B cells. However, by subdividing the effect of treatment in patients with active or not active disease we observed that disease activity rather than treatment was the major determinant affecting the proportions of DN1 and DN3 B cell subsets, a finding reinforced by data obtained after correction of the effect of treatment by multivariable analysis. It is noteworthy that antimalarial use was associated with increased frequencies of DN1 and reduced frequencies of DN3 B cells. Antimalarials are well known for reducing disease activity [48] and may affect B cell responses by inhibiting TLR7 signals [49]. Further prospective and mechanistic studies are needed to assess whether TLR7 inhibition induced by antimalarials would lead to lower disease activity by reducing the frequencies of DN3 B cells.

In our SLE female cohort following DN1, the frequency of DN3 cells was the second most frequent. The literature offers little evidence for the role of the DN3 subset in inflammatory disorders. An increased frequency of DN3 B cells with low expression of CXCR5 was reported in severe COVID-19 identified in CD11c- and CD21^−^ DN B cells [15,46], in autoimmune-fibrosis [18], in chronic cutaneous lupus [50], and increased early after Rituximab treatment in SLE [17]. The DN3 subset is proposed to have an extrafollicular origin [15,18] and, by exploiting single cell transcriptomics on a limited number of cell donors, suggested to take origin from a memory compartment in its way to acquire DN2 characteristics [51]. While we have not attempted to define further phenotypic and functional properties of the DN3 subset, the strong and distinct association in our SLE cohort with disease activity may point to an independent origin from the DN2 subset. This would be consistent with the individual transcriptional identity documented by bulk transcriptomics which allows a clear distinction between the DN2 and DN3 subsets [18]. Indeed, DN3 B cells were found to express a unique transcriptional signature and features of PB and ASCs suggestive of non-overlapping properties of DN3 B cells compared to other DN subsets [18]. Interestingly, tissue-infiltrating DN3 B cells have been reported in patients with dysregulated immunity and shown to interact with CD4^+^ T cells, suggesting that activated DN3 B cells could be drivers of pathology [18]. Whether DN3 B cells in the context of SLE can establish cognate interactions with CD4^+^ T cells in specific tissue and contributes to pathogenesis will warrant further investigations.

One of the reasons to assess TLR7 polymorphisms in our cohort was to test whether they could be associated with increased numbers of ABC cells. The hypothesis was that the G allele of snp rs3853839 could have enhanced the sensitivity of TLR7 to natural ligands therefore specifically enhancing the response of the ABC subset known to be particularly sensitive to TLR7 activation [13,35]. The findings however stress a potential larger role of the G snp rs3853839 allele in sustaining greater frequencies of total B cells accompanied by increase of CD11c-CXCR5+ follicular B cells, the numerically major B cell subset in SLE peripheral blood. These findings are consistent with the recently reported higher levels of TLR7 expression associated with the G allele and enhanced numbers of newly recruited transitional B cells [36]. Further studies are required to dig in the functional role of TLR7 polymorphisms in humans also taking into consideration the possibility of indirect effects on B cells mediated by other cell types.

Our work has some limitations. First, the individuals included in our cohort are representative of a prevalent SLE population under chronic treatment, which may hinder the identification of B cell subsets mostly involved at disease initiation and our phenotypic data have correlative and not causal relationship with disease characteristics. However, we were able to identify the DN3 B cell subset and CD19 MFI as strongly associated with disease activity independently from the effect of treatment as demonstrated by multivariable analysis (Table 1) and by the analysis of B cell subset frequencies in females not receiving individual therapeutic agents (Fig. S6). This approach allowed to disentangle the dynamic relationships occurring in the majority of SLE patients followed on the long term, in whom disease activity affects treatment intensity and treatment affects disease activity, by highlighting which B cell subsets were mostly affected by activity and treatment.

Second, in our study we had no longitudinal follow up and prospective studies should be performed to test whether the B cell subsets here identified as associated with disease activity, can also perform as valuable predictors.

Third, we have not attempted to assess whether the B cell subset abnormalities here identified apply only to SLE or are shared by other systemic autoimmune disorders. Further studies are needed to address this point.

Fourth, we used a very limited number of B cell markers to phenotypically characterize our SLE female cohort. However, despite limited characterization we identified both DN3 and CD19MFI as potential useful biomarkers. Finally, only females were included in our study, which is obvious limitation to the generalization of our findings. Indeed, males usually represent 10–20 % of SLE cohorts submitted to studies like ours and SLE outcome tend to be more severe in males [52]. Whether the B cell subset abnormalities here described are also present in males with SLE, would need specific studies.

In conclusion, by focusing on female SLE we have identified a relationship between the frequency of B cells belonging to the DN3 subset, their expression level of CD19 and disease clinical activity. Thus, our data contribute to the identification of DN3 B cells as potential targets of future therapeutic approaches.

SLE immunopathogenesis is complex and B cells play a key role in the disease process in SLE. Which aspect of B cell activities makes the biggest contribution to disease pathogenicity/activity remains controversial as alterations in all B cell compartments have been identified in SLE. Notably, both follicular (CXCR5+) and extrafollicular (CXCR5-) B cell responses are altered in SLE and in both TLR7 engagement plays a role [28]. Our data by showing the combined decreased percentage of CXCR5+ and decreased expression of CD19 - particularly in the double negative (CD27-IgD-) B cell subset - which associate with disease activity support the contribution of extrafollicular B cell expansion to SLE pathogenesis as elegantly proposed by others [53]. Further, the expanded DN3 B cell subset here identified may represent a novel therapeutic target.”

## Funding

This study was supported by the FOREUM 10.13039/501100014034Foundation for Research in Rheumatology, which had no role in the study design; in the collection, analysis and interpretation of data; in the writing of the report; and in the decision to submit the article for publication.

## CRediT authorship contribution statement

**Carlo Chizzolini:** Writing – original draft, Funding acquisition, Formal analysis, Data curation, Conceptualization. **Jean-Charles Guery:** Writing – review & editing, Project administration, Funding acquisition, Data curation, Conceptualization. **Fanny Noulet:** Writing – review & editing, Methodology, Investigation, Data curation. **Lyssia Gruaz:** Writing – review & editing, Investigation. **Claire Cenac:** Writing – review & editing, Methodology, Investigation. **Loredana Frasca:** Writing – review & editing, Funding acquisition, Conceptualization. **David Spoerl:** Writing – review & editing, Investigation. **Lionel Arlettaz:** Writing – review & editing, Investigation. **Alice Horisberger:** Writing – review & editing, Investigation, Data curation. **Camillo Ribi:** Writing – review & editing, Resources, Investigation, Data curation. **Stéphanie Hugues:** Writing – review & editing, Supervision, Funding acquisition, Formal analysis, Conceptualization.

## Declaration of competing interest

The authors declare the following financial interests/personal relationships which may be considered as potential competing interests:Jean_Charles Guery reports was provided by FOREUM. If there are other authors, they declare that they have no known competing financial interests or personal relationships that could have appeared to influence the work reported in this paper.

## Figures and Tables

**figs1:**
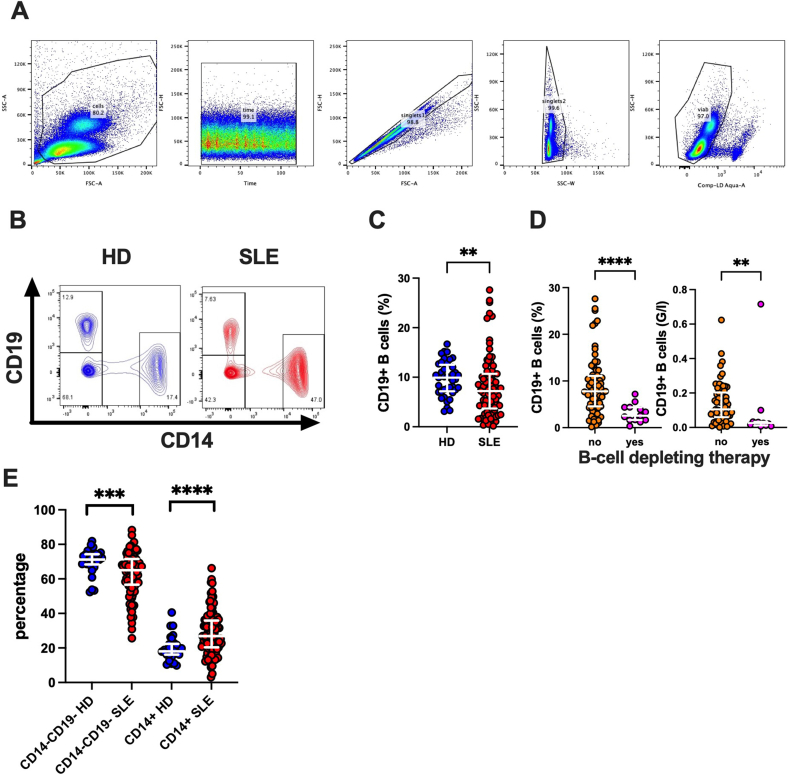


**figs2:**
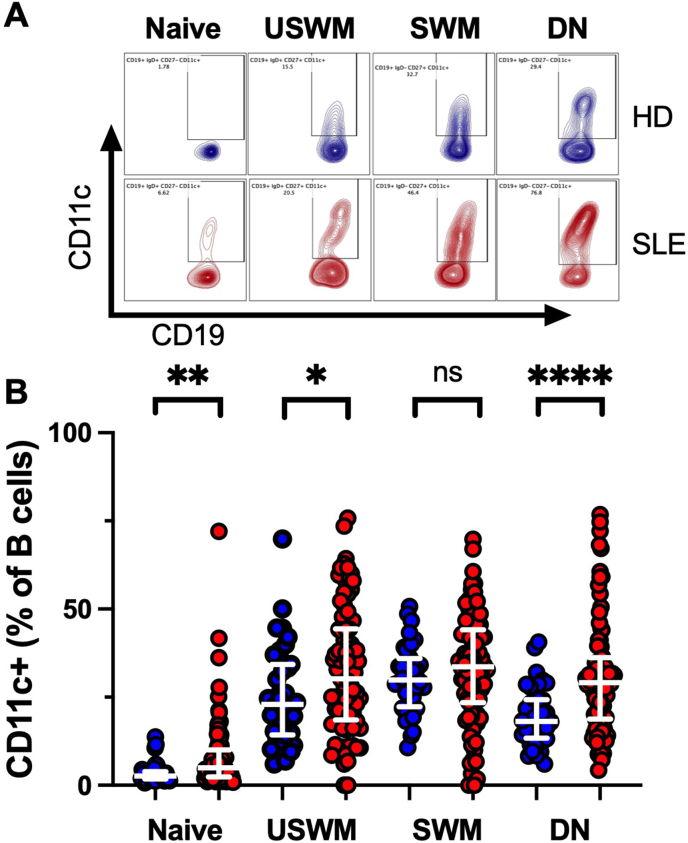


**figs3:**
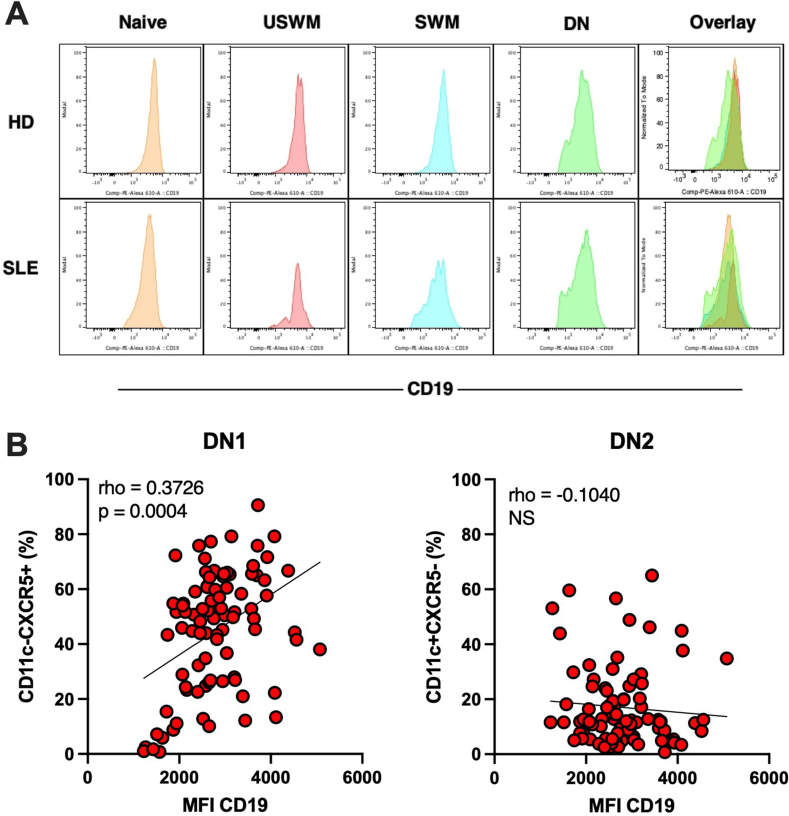


**figs4:**
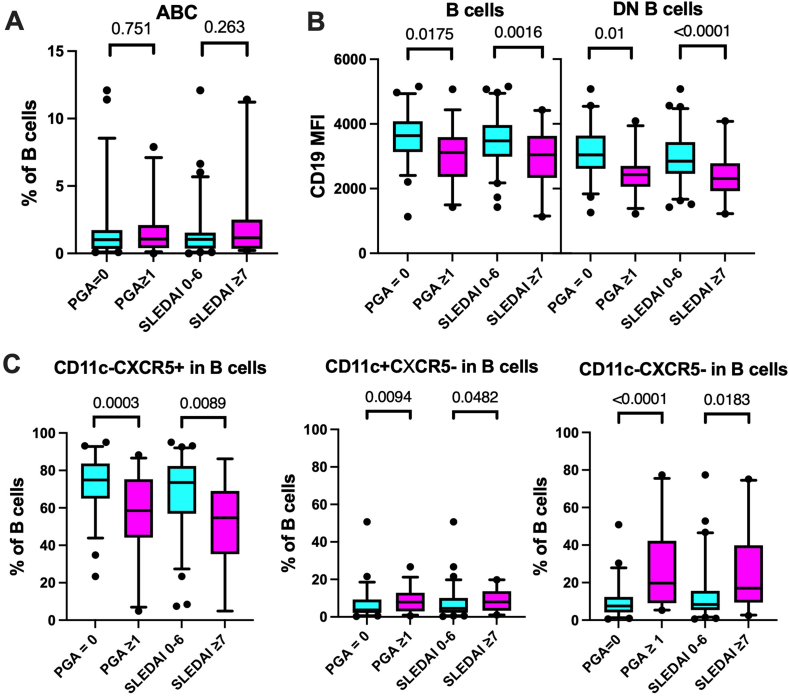


**figs5:**
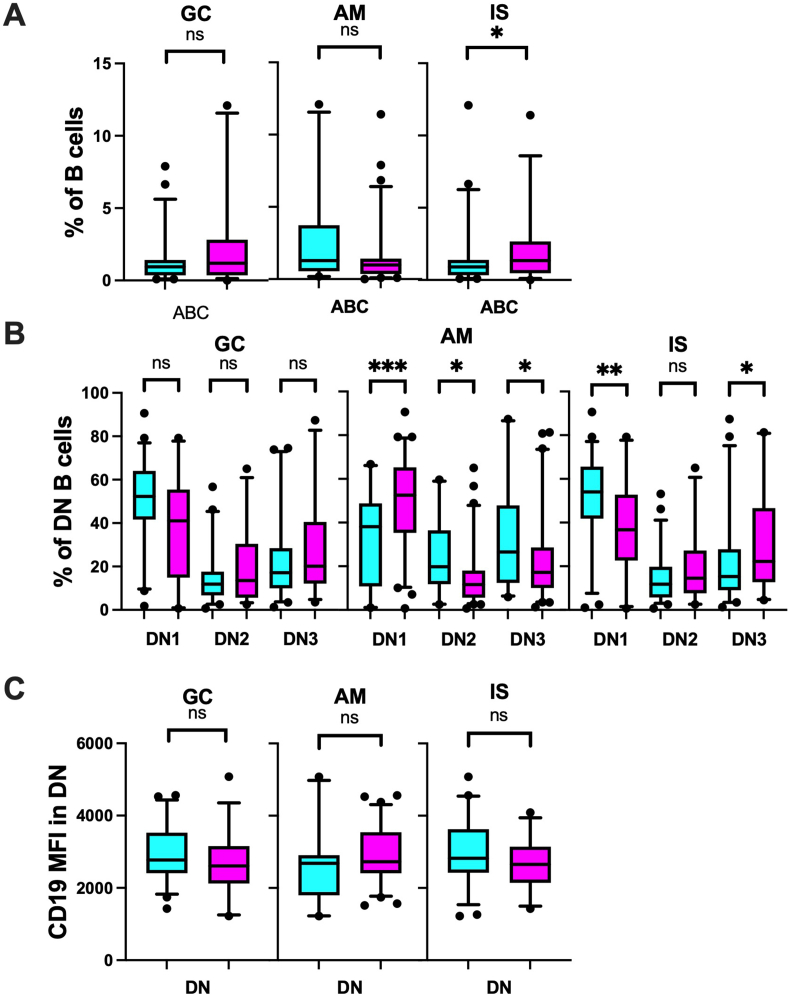


**figs6:**
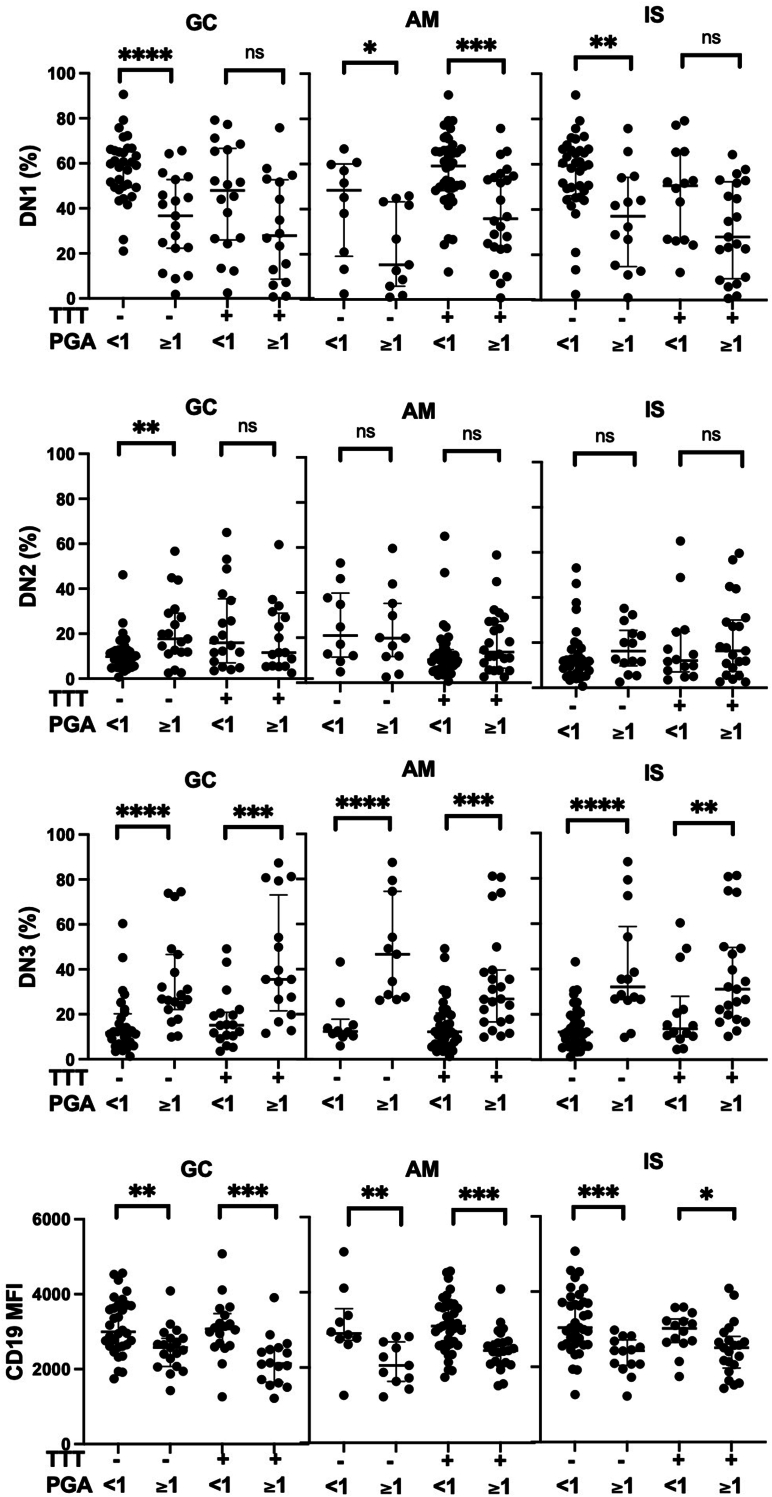


**figs7:**
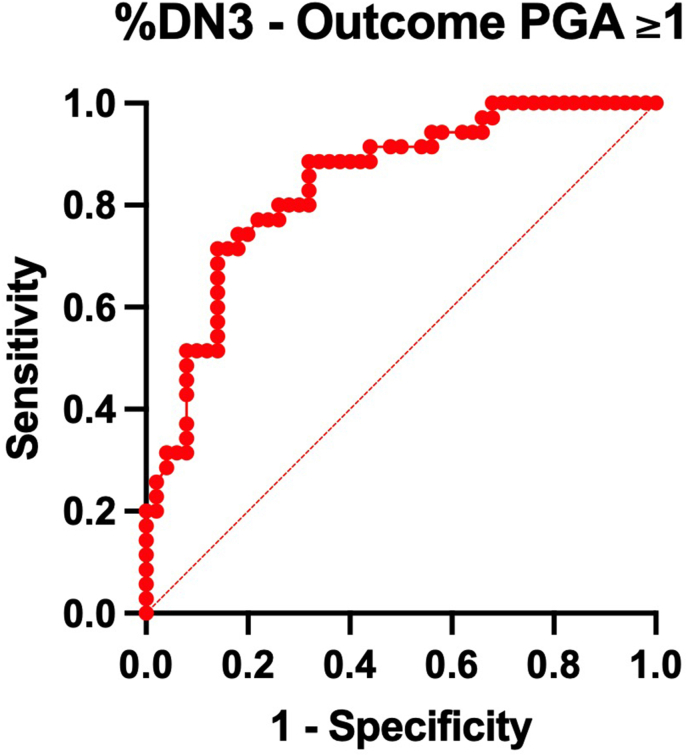


## Data Availability

Data will be made available on request.

## Abbreviations

AM: Antimalarial agents
AUC: Area under the curve
CXCR5: receptor of CXC-chemokine 5
DN: double negative
GC: Glucocorticoids
IQR: interquartile range
IS: Immunosuppressant agents
MFI: mean fluorescence intensity
PBMC: peripheral blood mononuclear cells
ROC: Receiving operator curve
SELENA: Safety of Estrogens in Lupus Erythematosus National Assessment
SLE: Systemic lupus erythematosus
SLEDAI: SLE disease activity index
Snp: single nucleotide polymorphism
SSCS: Swiss SLE Cohort Study
TLR7: Toll-like receptor 7
XCI: X-chromosome inactivation
XIST: X-inactive specific transcript
